# Association between *angiotensin converting enzyme* gene insertion/deletion polymorphism and renal scar risk in children vesicoureteral reflex: a reappraise meta-analysis

**DOI:** 10.1038/srep31243

**Published:** 2016-08-10

**Authors:** Jin-Wei Ai, Xian-Tao Zeng, Ying Liu, Yu Fu, Tong-Zu Liu, Bin Pei

**Affiliations:** 1 Evidence-Based Medicine Center, Xiangyang Hospital, Hubei University of Medicine, Xiangyang 441000, P.R. China; 2 Department of Urology, Zhongnan Hospital of Wuhan University, Wuhan 430071, P.R. China; 3 Center for Evidence-Based Medicine and Translational Medicine, Zhongnan Hospital of Wuhan University, Wuhan 430071, P.R. China; 4 Center for Evidence-Based Medicine and Translational Medicine, Wuhan University, Wuhan 430071, P.R. China; 5 Department of Pediatrics, Shiyan People’s Hospital, Hubei University of Medicine, Shiyan 442000, P.R. China

## Abstract

Vesicoureteral reflex(VUR) is a common disease in children. Some studies indicated that the *angiotensin converting enzyme* (*ACE*) gene insertion/deletion (I/D) polymorphism associated with the renal scar in VUR, but not all researchers agreed with it. To clarify the effect of *ACE* I/D polymorphism on renal scar risk in children with VUR, we performed the present meta-analysis. PubMed, CNKI, CBM, and Embase databases were searched for studies that examined the relationship between *ACE* I/D polymorphism and renal scar risk in children with VUR. The Stata 12.0 software was used for statistical analyses. 11 case-control studies with 1,032 VUR patients were analyzed. The results showed that the DD genotype and D allele were associated with renal scar risk in overall VUR patients, DD vs. DI + II: OR = 1.61, 95% CI = 1.04–2.49, *P* = 0.03; DD vs. II: OR = 1.78, 95% CI = 1.20–2.65, *P* < 0.01; D vs. I: OR = 1.38, 95% CI = 1.02–1.86, *P* = 0.04. Similar results were revealed in Turks, but not in Caucasians and Asians. Our meta-analysis indicated that the *ACE* DD genotype may increase the risk of renal scar in children with VUR.

Vesicoureteral reflex(VUR) is a common disease, with an incidence of 1–2% in children^1^. It is a common lower urinary tract malformation characterized by incompleteness of the junction between the ureter and bladder^2^. As a consequence of this malformation, retrograde urine stream flows from the bladder back into the ureter, pelvis, and medullary collecting ducts of the kidney, which may lead to progressive renal scarring, hypertension, recurrent urinary tract infections (UTI), tubulointerstitial renal disease, reflux nephropathy (RN), and chronic renal failure (CRF)^2^,^3^. Renal scar formation may be a key course through which VUR evolves to RN and CRF. Thus, the main goal of the current treatment of VUR is to protect renal function and prevent renal scar formation^4^,^5^. The pathogenesis of renal scar in VUR is multifactorial. In the past two decades, considerable studies have demonstrated that genetic factors play an important role in the VUR renal scar formation^3^. *Angiotensin converting enzyme* (*ACE*) gene insertion/deletion (I/D) polymorphism have been extensively investigated in this field.

The *ACE* gene is located on the chromosome 17q23, and contains 26 exons and 25 introns. Based on the presence or absence of a 287-base pair (bp) Alu repetitive sequence in the intron 16, it is divided as insertion/deletion (I/D) polymorphisms^6^. In other words, the gene possesses two alleles (D and I), and displays three genotypes (DD, DI and II). Previous studies have indicated that the *ACE* I/D polymorphism associated with the diabetic nephropathy^7^,^8^, IgA nephropathy^9^,^10^, nephritic syndrome^11^, focal segmental glomerulosclerosis^12^, autosomal dominant polycystic kidney disease^13^, etc. Recently, we have performed a meta-analysis on *ACE* I/D polymorphism and VUR risk, demonstrating that the DD genotype and D allele may increase the VUR risk in children^14^. However, regarding the question of whether it is a genetic susceptibility factor to renal scar formation in VUR, controversy and uncertainty remains in evidence from current studies. Some studies showed that the *ACE* DD genotype and D allele increased the risk of renal scar in VUR, with which not all researchers agree.

In 2012, a standard meta-analysis based on seven case-control studies was performed by Zhou *et al*.^15^ to identify the association between *ACE* I/D polymorphism and renal scar risk in VUR. However, this study has evident limitations such as limited sample size and apparent publication bias. Additional information from four new relevant studies published afterwards is currently available. Therefore, we carried out an updated meta-analysis with all eligible studies taken into consideration, in order to further clarify the association between the *ACE* I/D polymorphism and renal scar risk in children with VUR.

## Methods

This meta-analysis was reported in accordance with the PRISMA guidelines^16^.

### Inclusion and exclusion criteria

The study was considered eligible if it met the following criteria: (1) case-control or cohort study design; (2) the association of the *ACE* I/D polymorphism with renal scar in children VUR was investigated; (3) diagnostic imaging techniques such as renal ultrasonography, voiding cystourethrography, or nuclear scan with technetium-99m-dimercaptosuccinic acid (99m Tc-DMSA) was used for the diagnosis of VUR and renal scar; (4) VUR patients divided into two groups: with and without renal scar; and (5) provided were sufficient data (the numbers of DD, DI and II genotypes distribution in two groups, respectively) for calculating the odds ratio (OR) and it 95% confidence interval (CI). We excluded the editorials, brief reports, and duplicated data from multiple publications.

### Literature search

A comprehensive search was conducted to identify all eligible publications in PubMed, CNKI (China National Knowledge Infrastructure), CBM (China Biological Medicine Database), and Embase electronic databases up to 18 November 2015. The medical subject headings (MeSH) and free-text words were used. Search terms mainly included (“peptidyl-dipeptidase A”[MeSH] OR angiotensins OR “angiotensin converting enzyme” OR ACE) AND (“Polymorphism, Genetic”[MeSH] OR “genetic variation” OR “genetic polymorphism”) AND (“Vesico-Ureteral Reflux” [MeSH] OR “vesco-uretric reflux” OR “vesicoureteral reflux” OR VUR). The detailed search strategy is shown in Supplementary File S1. We also carefully checked references of the retrieved articles to find additional eligible studies. During the course of literature search, no language or other limits were set.

### Data extraction

Two investigators independently extracted the following items from each eligible study: Surname of first author, country of the investigation, year of publication, ethnicity, average age and genotype distribution of two groups, genotyping method, and the diagnostic approach for VUR and renal scar. A discussion was carried out to achieve consensus when discrepancy noted.

### Methodological quality assessment

Two investigators independently evaluated the quality of eligible studies using the Newcastle-Ottawa Scale^17^ (NOS), which was one of the most commonly used tools for assessing observational studies’ quality in a meta-analysis setting. The NOS encompasses three parts, ie case and control selection, comparability, and exposure. Each of them respectively comprises four, two, and three items. Each item is given 1 point, 9 points in total. If less than 7 points the study got, it would be regarded as “low quality”; otherwise would be regarded as “high quality”. A discussion was carried out to achieve consensus when discrepancy noted.

### Data analysis

Statistical analysis was performed using Stata12.0 software (Stata corporation, college station, TX, USA). The following genetic models were selected: DD vs. DI + II, DD + DI vs. II, DD vs. II, DI vs. II, and D vs. I. The strength of the association was expressed by odd ratio (OR) with its 95% confidence interval (CI). The *I*^2^ statistic and *Q* test were used to measure the between-study heterogeneity. If *I*^2^ < 50% and *P* *>* 0.1, the heterogeneity was considered mild, and the summary ORs were combined under a fixed-effects model, otherwise a random-effects model were used. The *Z* test was used to assess the statistical significance of pooled ORs, and two-tailed *P*-values < 0.05 were considered significant. Subgroup analysis stratified by ethnicity was conducted. Sensitivity analysis were performed by excluding the study with “low quality”, and excluding the study with the biggest OR outlier in genetic models with statistically significant findings. Visual inspection of funnel plots and Egger’s regression asymmetry test were applied to assess potential publication bias.

## Results

### Study selection

**Figure 1:**
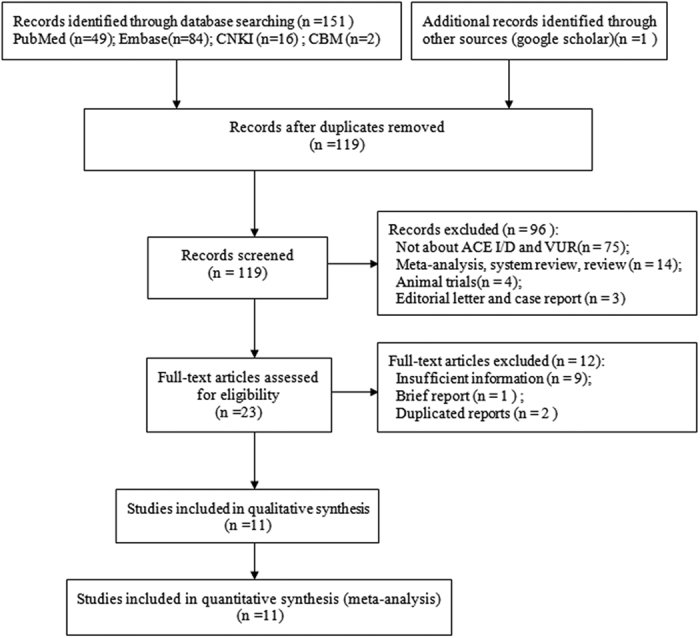
Flow diagram of the selection process for eligible studies.

Figure 1 shows our study selection details. 152 studies were preliminary retrieved. According to the inclusion criteria, 11 case-control studies^18^,^19^,^20^,^21^,^22^,^23^,^24^,^25^,^26^,^27^,^28^ with 1,032 VUR patients (561 with and 471 without renal scar) were included in our meta-analysis. Studies excluded during full-text screening are listed along with the reasons for exclusion in Supplementary File S2.

### Characteristics of included studies

All the eligible studies were issued in English, and investigated the association between *ACE* I/D polymorphism and renal scar risk in children VUR. Five studied^18^,^19^,^20^,^21^,^22^ in Caucasian, three^23^,^24^,^25^ in Asian, and three^26^,^27^,^28^ in Turkey. Table 1 presents the primary characteristics and quality assessment of the included studies. In all studies, diagnostic imaging techniques (such as renal ultrasonography, voiding cystourethrography) were used to confirm VUR, nuclear scan with technetium-99m-dimercaptosuccinic acid was used to detect renal scar, and polymerase chain reaction (PCR) technique was used for genotyping. The *ACE* D allele average frequency in VUR with renal scar group was higher than without renal scar group, and was highest in the Turkish renal scar group. Table 2 shows the average frequency of the D allele in each group. The quality of primary studies assessed by NOS. Three studies^20^,^23^,^28^ got 6 score, others more than 6. In other words, three studies were rated as “low quality”, and eight as “high quality”.

### Meta-analysis

Table 3 presents summary results concerning the association between *ACE* I/D polymorphism and the risk of renal scar in children with VUR.

**Figure 2:**
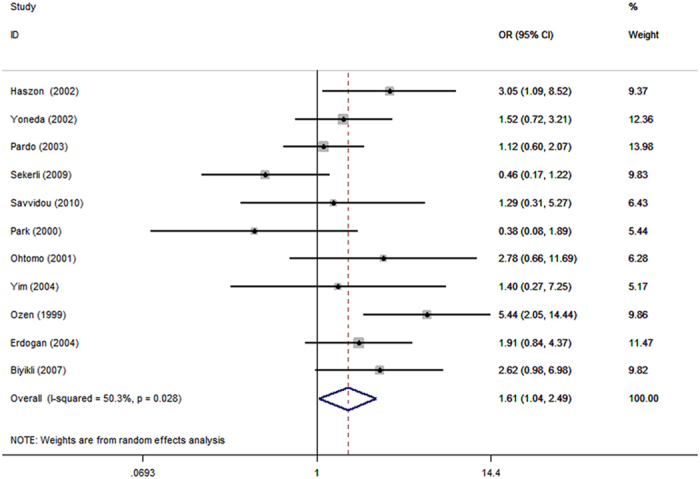
Forest plot of ACE I/D polymorphism and renal scar in children VUR DD vs. DI + II genetic model.

All studies investigated the association of *ACE* I/D polymorphism and renal scar risk in VUR. The pooled results manifested that the *ACE* DD genotype and D allele increased the risk of renal scar in VUR. DD vs. DI + II: OR = 1.61, 95% CI = 1.04–2.49, *P* = 0.03, Fig. 2; DD vs. II: OR = 1.78, 95% CI = 1.20–2.65, *P* < 0.01; and D vs. I: OR = 1.38, 95% CI = 1.02–1.86, *P* = 0.04.

Results of subgroup analyses stratified by ethnicity suggested that there were significant positive association between the DD genotype and D allele and renal scar susceptibility in Turks. DD vs. DI + II: OR = 2.89, 95% CI = 1.57–5.32, *P* < 0.01; DD vs. II: OR = 4.30, 95% CI = 1.68–10.98, *P* < 0.01; and D vs. I: OR = 2.08, 95% CI = 1.43–3.02, *P* < 0.01. But we did not detect any significant associations between *ACE* I/D polymorphism and renal scar in Caucasians and Asians.

### Sensitivity analysis

**Figure 3:**
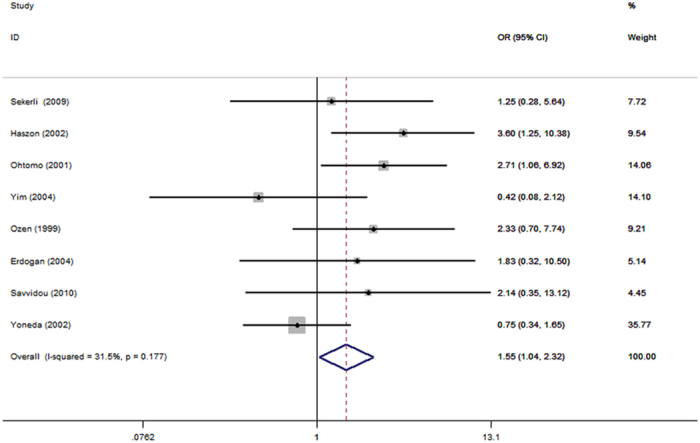
Sensitivity analysis. Performed by excluding the “low quality” studies, DD + DI vs. II genetic model.

Sensitivity analysis performed by excluding the “low quality” studies^20^,^23^,^28^ which got an NOS score < 7, Table 4. The results showed that the pooled results were not significant changed for all genetic models, except for the DD + DI vs. II in the overall population, DD + DI vs. II: OR = 1.55, 95% CI = 1.04–2.32, *P* = 0.03, Fig. 3.

**Figure 4:**
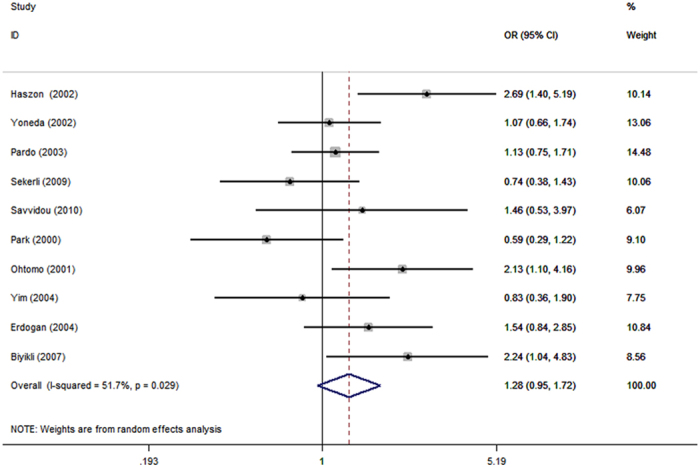
Sensitivity analysis. Performed by excluding the study with the biggest OR outlier, D vs. I genetic model.

We also took another sensitivity analysis by excluding the study^26^ with the biggest OR outlier in the three genetic models with statistical significant findings. Because the study came from Turkey, we recalculated the pooled effects of the Turkish subgroup, Table 5. We found the pooled results were significantly changed in two genetic models, D vs. I and DD vs. II in the overall population group and Turkish subgroup, respectively. All of them became having no statistical significance. D vs. I: OR = 1.28, 95% CI = 0.95–1.72, *P* = 0.10, Fig. 4, and DD vs. II: OR = 3.21, 95% CI = 0.91–11.35, *P* = 0.07.

### Publication bias

**Figure 5:**
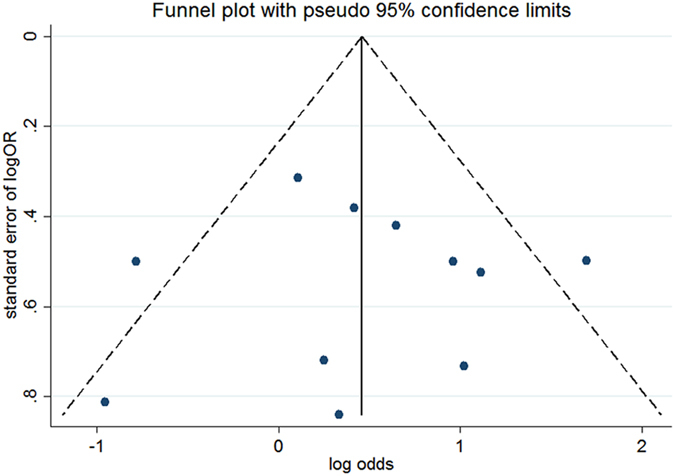
Funnel plot to detect publication bias DD vs. DI + II genetic model.

Visual inspection of funnel plots and Egger’s test were used to evaluate the publication bias in our meta-analysis. The shape of the funnel plots seems symmetrical. Taking the DD vs. DI + II genetic model as an example, the funnel plot was displayed in Fig. 5. The statistical results still showed there were no publication bias in our study (Table 3).

## Discussion

The association between *ACE* I/D polymorphism and the renal scar risk in children with VUR remains controversial. The inconsistent results may be attributed to small sample size in single investigation, and ethnic variation existing among studies’ populations, etc. A meta-analysis, performed by Zhou *et al*.^15^, suggested that the *ACE* I/D polymorphism was not correlated with the risk of renal scar in the overall population. However, the sample size was still relatively small. Accordingly, in order to further clarify the association of *ACE* I/D polymorphism with the renal scar risk in children with VUR, we included all eligible studies for increasing the sample size and statistical efficiency, and providing more reliable conclusions. The pooled results showed that the *ACE* DD genotype increased the risk of renal scar in overall study population. Subgroup analyses stratified by ethnicity, the results indicated that DD genotype and D allele increased the risk of renal scar in Turks. Although there were no statistical significance in five genetic models in Caucasians, the DD genotype and D allele had a tendency to increase the renal scar risk (all *ORs* >1). But the association and tendency did not exist in Asians.

The DD genotype and D allele were risk factors for renal scar in Turks, but the association was not obvious in Caucasians and Asians. The D allele average frequency and the DD genotype percentage, which were the highest in Turks, could contribute to the difference (Table 2). It showed that racial difference existed between *ACE* I/D polymorphism and the renal VUR renal scarring risk, in addition, the higher the D allele average frequency and the DD genotype percentage in VUR population, the greater renal scar morbidity. So, the D allele carriers in VUR may took on more risk in renal scar formation.

In the sensitivity analysis, when we excluded the low quality studies, the result of DD + DI vs. II genetic model significantly changed in the overall population group, but others had no obvious change, especially in DD vs. DI + II, DD vs. II, and D vs. I genetic model. This change did not affect our final conclusions, and supported our findings. Because it implied that the D allele carriers in VUR may have a higher risk of renal scar formation, DD + DI vs. II: OR = 1.55, *P* = 0.03.

We also performed a sensitivity analysis by excluding the study with the biggest OR outlier in genetic models with statistically significant findings. Significant change in pooled results occurred with two genetic models, ie D vs. I in the overall population group, and DD vs. II in the Turkish subgroup. It indicated that the conclusion of D allele relative to I in the overall population, and DD genotype relative to II in the Turkish increasing the risk of renal scar were not stability enough, further researches are needed to verify it. From the two sensitivity analyses, we can find that the results of DD vs. DI + II in all groups, and D vs. I in the Turkish subgroup were not significant changed. So the conclusion of DD genotype increased the risk of renal scar formation in children VUR, and DD genotype and D allele increased the risk in Turkey were credible. In addition, our study did not have publication bias. Thus, our conclusions were robust.

Although the association between the *ACE* I/D polymorphism and kidney diseases has been extensively researched, there was still obviously controversy about whether the DD genotype and/or D allele increase the renal scar susceptibility in VUR. Most studies suggest that there was no association between *ACE* I/D gene polymorphism and VUR renal scar, only a few studies have shown a positive association between them. The controversial conclusions may mainly be caused by ethnic variation, and different ethnic origin may have disparate genetic background^29^,^30^. It may be primary source of heterogeneity in our meta-analysis. Subgroup analysis showed that significant associations between *ACE* I/D polymorphism and renal scar risk only existed in Turkey, which can be partly interpreted by the higher average frequency of D allele in the Turkish VUR patients than other subgroups (Table 2). The heterogeneity among the studies were minimal, *I*^2^ = 0% in all genetic comparison models, except for in DD + DI vs. II model (*I*^2^ = 23.5%), and the pooled results showed strongly positive correlation (OR_DD vs. II+DI_ = 2.90, OR_DD vs. II_ = 4.30, OR_D vs. I_ = 2.08). In Caucasians, the heterogeneity was also decreased significantly, with *I*^2^ < 50.0% in all genetic comparison models, except for in D vs. I (*I*^2^ = 52.3%). However, in Asians, the heterogeneity presented in some genetic models, owe to the limited data we failed to further detect the source of the heterogeneity.

All the subjects in those studies, which included in our meta-analysis, were VUR patients. In most of the studies, renal scar was detected by 99m Tc-DMSA, and VUR accompanied with UTI, hypertension, ureteropelvic junction obstruction, posterior urethral valves, and CRF, etc. So, Renal scar can be secondary to VUR or other concomitant diseases. However, the studies did not report the distribution of *ACE* I/D genotypes in different pathogeneses of scar formation. Because of insufficient information, we did not analysis the association between the genetic polymorphism and renal scar in different pathogeneses.

The mechanism through which *ACE* DD genotype and D allele increased the risk of renal scar formation in VUR are still unclear^31^. Previous studies demonstrated that *ACE* DD genotype enhanced the ACE expression. The DD genotype had association the highest ACE level^32^. As we all know that ACE is a key enzyme in the renin-angiotensin system, and its mainly function is to converse the Angiotensin I (Ang I) to Angiotensin II (Ang II). The latter is the most powerful factor in blood-pressure regulation, cardiovascular function accommodation, and electrolyte homeostasis^30^. Besides, the Ang II was considered as a growth factor that plays an important role in the pathology of kidney diseases^33^. Possible evidence as follows, firstly, the concentrations of Ang II in local kidney and interstitial fluid are greater than circulating levels. Secondly, blockade of the actions of Ang II delay kidney functional and structural deterioration. Thirdly, the renal lesions can be reduced by treatment with angiotensin I-converting enzyme inhibitors and angiotensin receptor blockers^25^,^26^. In addition, Ang II takes part in the progression of renal disease through hemodynamic effects, growth-related and prosclerotic effects^25^. Therefore, the *ACE* DD genotype and D allele may link to ACE and Ang II to accelerate VUR renal scar formation. However, more studies should be carried out to elucidate the precise pathophysiologic mechanisms of the *ACE* DD genotype and D allele increasing the renal scar risk in children VUR.

In 2012, Zhou *et al*.^15^ performed a meta-analysis to investigate the association between *ACE* I/D polymorphism and renal scar risk in VUR. Seven studies were included in this study (four Caucasian, one Asian and two Turkish), which indicated that the *ACE* I/D polymorphism was not related to the risk of renal scar in the overall population, but the DD genotype and D allele increased the risk in Turks. The study^15^ sample size is relatively small, and has obviously publication bias. Therefore, we performed the updated meta-analysis including more eligible studies to reappraise the association. We found that the DD genotype increased the risk of renal scar in the overall study population, and this finding is inconsistent with that of Zhou *et al*.^15^. In the subgroup analysis, our finding is the same as Zhou *et al*.^15^, which has further confirmed that the DD genotype and D allele are risk factors for renal scar formation in Turkish VUR patients. The number of included studies and the sample size in our meta-analysis are larger than Zhou *et al*.^15^. In addition, our study had no obvious publication bias. Thus, our conclusions were more reliable.

Our study had some limitations. First, we did not account for some confounding factors, such as gender, family history and environment, etc. Second, there was mild-moderate heterogeneity across included studies. Although we performed a subgroup analysis by ethnicity, obvious heterogeneity still existed in some genetic models, and we failed to explain the heterogeneity completely. Third, the sample sizes in some studies were relatively small, thus due to lack of sufficient power, it may overestimate the true association. Forth, our study was also limited by lack of the race of African. Last but not least, due to the insufficient information, we did not analysis the association between the genetic polymorphism and renal scar in different pathogeneses. Future studies should better focus on this aspect.

## Conclusions

Our meta-analysis demonstrated that *ACE* DD genotype may increase the risk of renal scar in children with VUR. However, the association between *ACE* I/D polymorphism and renal scar risk had ethnic variations. The DD genotype and D allele may increase the risk of renal scar in Turks, but not in Caucasians and Asians. Due to the defects of the original research, more large-scale investigations with appropriate design are required to further verify our findings.

## Additional Information

**How to cite this article**: Ai, J.-W. *et al*. Association between *angiotensin converting enzyme* gene insertion/ deletion polymorphism and renal scar risk in children vesicoureteral reflex: a reappraise meta-analysis. *Sci. Rep*. **6**, 31243; doi: 10.1038/srep31243 (2016).

## Supplementary Material

Supplementary Information

## Figures and Tables

**Table 1:** Characteristics of the studies included in this meta-analysis.

Author	Year	Country	Ethnicity	Sample Size	Geno-typing	VUR Scar(+)				VUR Scar(-)				NOS Score
						DD	DI	II	D%	DD	DI	II	D%	
Haszon	2002	USA	Caucasian	77	PCR	19	17	7	64.0%	7	13	14	39.7%	8
Yoneda	2002	Ireland	Caucasian	162	PCR	15	18	12	53.3%	29	63	25	51.7%	7
Pardo	2003	Spain	Caucasian	206	PCR	47	69	21	59.5%	22	34	13	56.5%	6
Sekerli	2009	Greece	Caucasian	85	PCR	13	39	5	57.0%	11	14	3	64.3%	7
Savvidou	2010	Greece	Caucasian	33	PCR	6	6	2	64.3%	7	7	5	55.3%	9
Park	2000	Korea	Asian	66	PCR	3	22	17	33.3%	4	14	6	45.8%	6
Ohtomo	2001	Japan	Asian	78	PCR	7	20	11	44.5%	3	16	21	27.5%	7
Yim et	2004	Korea	Asian	67	PCR	10	28	15	45.3%	2	10	2	50.0%	7
Ozen	1999	Turkish	Turkey	94	PCR	28	20	5	71.7%	7	26	8	48.8%	7
Erdogan	2004	Turkish	Turkey	96	PCR	22	21	2	72.2%	17	30	4	62.8%	7
Biyikli	2007	Turkish	Turkey	68	PCR	22	10	2	79.4%	14	15	5	63.2%	6

PCR: polymerase chain reaction; VUR, vesico-ureteral reflux; Scar(+), with renal scar; Scar(−), without renal scar; NOS, Newcastle-Ottawa Scale.

**Table 2:** D% and DD% in different groups.

Group	Total		Turkish		Caucasian		Asian	
	D%	DD%	D%	DD%	D%	DD%	D%	DD%
VUR	55.3%	30.5%	66.3%	42.6%	51.2%	31.3%	39.8%	13.7%
Scar(+)	58.0%	34.2%	73.9%	54.5%	59.0%	33.8%	41.4%	15.0%
Scar(−)	51.8%	26.1%	58.3%	30.1%	42.7%	28.5%	37.2%	11.5%

VUR, vesico-ureteral reflux; Scar(+), with renal scar; Scar(−), without renal scar.

**Table 3:** A summary of the meta-analysis and subgroup analysis.

Genetic model	Group	N†	Heterogeneity test		Egger’s test(*P*)	Model selected	OR 95% CI	***P***
			***I***^**2**^	***P***				
DD vs. DI + II	Total	11	50.3%	0.03	0.96	Random	1.61 (1.04–2.49)	0.03
	Caucasian	5	45.6%	0.12	—	Fixed	1.23 (0.85–1.80)	0.27
	Asian	3	39.8%	0.19	—	Fixed	1.24 (0.53–2.92)	0.62
	Turkish	3	23.5%	0.27	—	Fixed	2.90 (1.72–4.89)	<0.01
DD + DI vs. II	Total	11	31.4%	0.15	0.70	Fixed	1.37 (0.99–1.91)	0.06
	Caucasian	5	29.8%	0.22	—	Fixed	1.36 (0.87–2.12)	0.18
	Asian	3	71.3%	0.03	—	Random	0.90 (0.25–3.24)	0.87
	Turkish	3	0.0%	0.95	—	Fixed	2.29 (0.97–5.41)	0.06
DD vs. II	Total	11	40.2%	0.08	0.84	Fixed	1.78 (1.20–2.65)	<0.01
	Caucasian	5	27.7%	0.24	—	Fixed	1.54 (0.94–2.55)	0.09
	Asian	3	66.2%	0.05	—	Random	0.98 (0.17–5.81)	0.98
	Turkish	3	0.0%	0.73	—	Fixed	4.30 (1.68–10.98)	<0.01
DI vs. II	Total	11	4.6%	0.40	0.72	Fixed	1.17 (0.82–1.66)	0.39
	Caucasian	5	16.3%	0.31	—	Fixed	1.21 (0.75–1.95)	0.44
	Asian	3	63.0%	0.07	—	Random	0.88 (0.27–2.83)	0.83
	Turkish	3	0.0%	0.96	—	Fixed	1.37 (0.56–3.36)	0.49
D vs. I	Total	11	58.2%	0.01	0.76	Random	1.38 (1.02–1.86)	0.04
	Caucasian	5	52.3%	0.08	—	Random	1.24 (0.84–1.84)	0.27
	Asian	3	71.6%	0.03	—	Random	1.03 (0.46–2.30)	0.94
	Turkish	3	0.0%	0.45	—	Fixed	2.08 (1.43–3.02)	<0.01

OR: odds ratio; CI: confidence interval.

^†^N: Number of studies.

**Table 4:** Sensitivity analysisΔ.

Genetic model	Group	N†	Heterogeneity test		Egger’s test(***P***)	Model selected	OR 95% CI	*P*
			***I***^**2**^	***P***				
DD vs. DI + II	Total	8	51.4%	0.05	0.95	Random	1.81 (1.05–3.10)	0.03
	Caucasian	4	58.4%	0.07	—	Random	1.28 (0.59–2.81)	0.53
	Asian	2	0.0%	0.54	—	Fixed	2.07 (0.70–6.12)	0.19
	Turkish	2	61.1%	0.11	—	Random	3.11 (1.12–8.67)	0.03
DD + DI vs. II	Total	8	31.5%	0.18	0.92	Fixed	1.55 (1.04–2.32)	0.03
	Caucasian	4	47.2%	0.13	—	Fixed	1.39 (0.81–2.42)	0.23
	Asian	2	74.1%	0.05	—	Random	1.21 (0.20–7.42)	0.84
	Turkish	2	0.0%	0.82	—	Fixed	2.14 (0.80–5.80)	0.13
DD vs. II	Total	8	32.8%	0.17	0.90	Fixed	2.15 (1.32–3.51)	<0.01
	Caucasian	4	44.0%	0.15	—	Fixed	1.68 (0.90–3.11)	0.10
	Asian	2	50.6%	0.16	—	Random	1.99 (0.32–12.54)	0.46
	Turkish	2	0.0%	0.44	—	Fixed	4.46 (1.47–13.46)	<0.01
DI vs. II	Total	8	18.3%	0.29	0.78	Fixed	1.25 (0.81–1.92)	0.31
	Caucasian	4	37.0%	0.19	—	Fixed	1.19 (0.65–2.15)	0.56
	Asian	2	72.5%	0.06	—	Random	1.07 (0.17–6.51)	0.95
	Turkish	2	0.0%	0.91	—	Fixed	1.28 (0.46–3.60)	0.63
D vs. I	Total	8	55.9%	0.03	0.99	Random	1.49 (1.05–2.13)	0.03
	Caucasian	4	63.5%	0.04	—	Random	1.30 (0.75–2.27)	0.35
	Asian	2	66.9%	0.08	—	Random	1.37 (0.55–3.47)	0.50
	Turkish	2	34.9%	0.22	—	Fixed	2.03 (1.18–1.86)	<0.01

OR: odds ratio; CI: confidence interval.

^Δ^Sensitivity analysis performed by excluding the low quality studies (NOS score <7).

^†^N: Number of studies.

**Table 5:** Sensitivity analysisΔ.

Genetic model	Group	N†	Heterogeneity test		Egger’s test(P)	Model selected	OR 95% CI	***P***
			***I***^**2**^	***P***				
DD vs. DI + II	Total	10	32.7%	0.15	0.96	Fixed	1.41 (1.04–1.90)	0.03
	Turkish	2	0.0%	0.63	—	Fixed	2.18 (1.16–4.09)	0.02
DD + DI vs. II	Total	10	34.8%	0.13	0.76	Fixed	1.31 (0.93–1.85)	0.12
	Turkish	2	0.0%	0.74	—	Fixed	2.26 (0.67–7.67)	0.19
DD vs. II	Total	10	31.7%	0.16	0.90	Fixed	1.59 (1.05–2.41)	0.03
	Turkish	2	0.0%	0.75	—	Fixed	3.21 (0.91–11.35)	0.07§
DI vs. II	Total	10	14.1%	0.31	0.74	Fixed	1.16 (0.81–1.68)	0.42
	Turkish	2	0.0%	0.89	—	Fixed	1.52 (0.43–5.47)	0.52
D vs. I	Total	10	51.7%	0.03	0.74	Random	1.28 (0.95–1.72)	0.10§
	Turkish	2	0.0%	0.46	—	Fixed	1.78 (1.11–2.88)	0.02

OR: odds ratio; CI: confidence interval.

^Δ^Sensitivity analysis by excluding the study with the biggest OR outlier.

^†^N: Number of studies.

^§^The pooled results were changed in sensitivity analysis.
