# Supplementary material for: Association between angiotensin converting enzyme gene insertion/deletion polymorphism and renal scar risk in children vesicoureteral reflex: a reappraise meta-analysis
Source: Sci Rep. 2016 Aug 10;6:31243. doi: 10.1038/srep31243 (PMC4978959; doi:10.1038/srep31243)
Supplement: Supplementary Information [file srep31243-s1.doc]

**Association between *angiotensin converting enzyme* gene insertion/ deletion polymorphism and renal scar risk in children vesicoureteral reflex: a reappraise meta-analysis**

Jin-Wei Ai, Xian-Tao Zeng, Ying Liu, Yu FU, Tong-Zu Liu, Bin Pei

**Supplementary File S1. The detailed search strategy in PubMed**

#1 Search "Vesico-Ureteral Reflux"[Mesh]

#2 Search (((((("vesico-ureteral reflux") OR "vesico ureteral reflux") OR "vesco-uretric reflux") OR "vesco uretric reflux") OR "reflux nephropathy") OR "vesicoureteral reflux") OR VUR

#3 #1 OR #2

#4 Search "Peptidyl-Dipeptidase A"[Mesh]

#5 Search (((Angiotensin) OR Angiotensins) OR "angiotensin converting enzyme") OR ACE

#6 #4 OR #5

#7 Search "Polymorphism, Genetic"[Mesh]

#8 Search (((((("Genetic Polymorphism") OR "Genetic Polymorphisms") OR "Genetic Variation") OR polymorphism) OR polymorphisms) OR gene) OR genome

#9 #7 OR #8

#10 #3 AND #6 AND #9 (Outcome:49)

**The complete search strategy:**  (((("Vesico-Ureteral Reflux"[Mesh]) OR ((((((("vesico-ureteral reflux") OR "vesico ureteral reflux") OR "vesco-uretric reflux") OR "vesco uretric reflux") OR "reflux nephropathy") OR "vesicoureteral reflux") OR VUR))) AND (("Peptidyl-Dipeptidase A"[Mesh]) OR ((((Angiotensin) OR Angiotensins) OR "angiotensin converting enzyme") OR ACE))) AND (("Polymorphism, Genetic"[Mesh]) OR ((((((("Genetic Polymorphism") OR "Genetic Polymorphisms") OR "Genetic Variation") OR polymorphism) OR polymorphisms) OR gene) OR genome))

**Supplementary File S2. List of the excluded studies with its reason during** **full-text screening**

| **NO**. | **Study citation** | **Reason for exclusion** |
| --- | --- | --- |
| 1. | Cho S, Lee S. ACE gene polymorphism and renal scar in children with acute pyelonephritis. *Pediatr Nephrol.* 2002;17(7):491-495. | Insufficient information about ACE I/D genotypes |
| 2. | Bajpai M, Pratap A, Somitesh C, Tyagi J. Angiotensin Converting Enzyme Gene Polymorphism in Asian Indian Children With Congenital Uropathies. *J Urol.* 2004;171(2):838-840. | Insufficient information about ACE I/D genotypes |
| 3. | Hohenfellner K, Wingen AM, Nauroth O, Wuhl E, Mehls O, Schaefer F. Impact of ACE I/D gene polymorphism on congenital renal malformations. *Pediatr Nephrol.* 2001;16(4):356-361. | Insufficient information about ACE I/D genotypes |
| 4. | Hohenfellner K, Hunley TE, Brezinska R, et al. ACE I/D gene polymorphism predicts renal damage in congenital uropathies. *Pediatr Nephrol.* 1999;13(6):514-518. | Duplicated reports |
| 5. | Liu K, Lin C, Chen H, Wei C, Lee C, Guey J. Renin-angiotensin system polymorphisms in Taiwanese primary vesicoureteral reflux. *Pediatr Nephrol.* 2004;19(6):594-601. | Insufficient information about ACE I/D genotypes |
| 6. | Hahn H, Ku S, Kim K, Park Y, Yoon C, Cheong H. Implication of genetic variations in congenital obstructive nephropathy. *Pediatr Nephrol.* 2005;20(11):1541-1544. | Insufficient information about ACE I/D genotypes |
| 7. | Akman B, Tarhan C, Arat Z, Sezer S, Ozdemir FN. Renin-angiotensin system polymorphisms: A risk factor for progression to end-stage renal disease in vesicoureteral reflux patients. *Renal Failure.* 2009;31(3):196-200. | Insufficient information about ACE I/D genotypes |
| 8. | Erdogan H, Ertan P, Ozkayin N, et al. ACE gene polymorphism and renal scarring in children with vesicouretheral reflux. *BANTAO Journal.* 2003;2(1):120-122. | No detail of with and without renal scar in VUR |
| 9. | Kowalewska P, Mynarski W, Modkowska E, *et al*. ACE gene polymorphism and renal scarring in children with urinary tract infection and vesicoureteric reflux - Rreliminary results. *Pediatria Polska.* 2003;80:102-105. | Duplicated reports |
| 10. | Kowalewska P, Mynarski W, Kubryn I, Bodalski J. Angiotensin-converting enzyme gene polymorphism and primary vesicoureteric reflux in children - One center study*.* Vol 2; 2004:121-125. | No detail of with and without renal scar in VUR |
| 11 | Dudley J, Johnston A, Gardner A, et al. The deletion polymorphism of the ACE gene is not an independent risk factor for renal scarring in children with vesico-ureteric reflux. *Nephrol Dial Transplant*. 2002; 17: 652-654. | Brief report |
| 12. | Dumlupynar Y, Cankorkmaz L, Koyluoolu G, *et al*. The relation between angiotensin converting enzyme gene polymorphism and renal scarring with vesicoureteral reflux. *Erciyed Medical Journal.* 2010;3(32):177-182. | No detail of with and without renal scar in VUR |
